# *Plasmodium berghei*-induced malaria decreases pain sensitivity in mice

**DOI:** 10.4102/ojvr.v88i1.1871

**Published:** 2021-01-11

**Authors:** Aboyeji L. Oyewole, Oluwole Akinola, Bamidele V. Owoyele

**Affiliations:** 1 Department of Physiology, Neuroscience and Inflammation Unit, College of Health Sciences, Faculty of Basic Medical Sciences, University of Ilorin, Ilorin, Nigeria; 2 Bioresearch Hub Laboratory, Ilorin, Nigeria; 3 Department of Anatomy, College of Health Sciences, Faculty of Basic Medical Sciences, University of Ilorin, Ilorin, Nigeria

**Keywords:** inflammatory pain, malaria, infection pain, behaviour, opioidergic, serotoninergic

## Abstract

Various types of pain were reported by people with *Plasmodium falciparum* and were mostly attributed to a symptom of malarial infection. Neural processes of pain sensation during malarial infection and their contributions to malaria-related death are poorly understood. Thus, these form the focus of this study. Swiss mice used for this study were randomly divided into two groups. Animals in the first group (*Pb*-infected group) were inoculated with *Plasmodium berghei* to induce malaria whilst the other group (intact group) was not infected. Formalin test was used to assess pain sensitivity in both groups and using various antagonists, the possible mechanism for deviation in pain sensitivity was probed. Also, plasma and brain samples collected from animals in both groups were subjected to biochemical and/or histological studies. The results showed that *Pb*-infected mice exhibited diminished pain-related behaviours to noxious chemical. The observed parasite-induced analgesia appeared to be synergistically mediated via µ-opioid, α2 and 5HT2A receptors. When varied drugs capable of decreasing pain threshold (pro-nociceptive drugs) were used, the survival rate was not significantly different in the *Pb*-infected mice. This showed little or no contribution of the pain processing system to malaria-related death. Also, using an anti-CD68 antibody, there was no immunopositive cell in the brain to attribute the observed effects to cerebral malaria. Although in the haematoxylin and eosin-stained tissues, there were mild morphological changes in the motor and anterior cingulate cortices. In conclusion, the pain symptom was remarkably decreased in the animal model for malaria, and thus, the model may not be appropriate for investigating malaria-linked pain as reported in humans. This is the first report showing that at a critical point, the malaria parasite caused pain-relieving effects in Swiss mice.

## Introduction

The World Malaria Report 2018 estimated the global tally of new malaria cases and death as at 2016 to be 216 000 000 and 445 000, respectively (WHO 2018). Despite several up-scaling interventions, increased hotspots for malaria transmission, zoonotic malaria transmission, emergence and re-emergence of resistant *Plasmodium* parasites have continued to amplify malaria prevalence globally.

At a specific parasitic density, nociceptors are either depolarised, hyperpolarised or sensitised, and this consequently results in a localised or generalised alteration in pain perception. Whilst the previous infection inflammation-induced pain dogma still holds, recent findings showed certain pathogens and their products can directly activate nociceptors. In the infection inflammation-induced pain pathway, pathogens and their products are known to have prototypic molecular pattern that stimulate Toll-like receptor which in turn activate biosynthesis and release of pro-inflammatory cytokines (Miller et al. 2009; Schedlowski, Engler & Grigoleit 2014). Apart from the cytokines (interleukins, tumour necrosis factor and neurotrophins) (Burnstock 2009), mediators like prostaglandins, histamine, purines, serotonin and bradykinins are also released primarily to recruit immune cells to the site of injury or infection. However, peripheral nociceptor terminals via their receptors and ion channels are sensitised by these mediators, consequently resulting in increasing pain sensation (Pinho-ribeiro et al. 2017). Although this pathway was believed for years as the sole genesis of pain during infection, emerging studies, however, showed a direct pathogen-induced pain at a specific density in parasitaemic infection.

In bacterial infection, for instance, lipopolysaccharide via its lipid A anchored moiety component can directly stimulate transient receptor potential ankyrin 1 ion channel on nociceptors to produce pain (Kashem et al. 2015). Also, a gram-positive bacterium like *Staphylococcus aureus* produces α-haemolysin that stimulates nociceptors. This forms the basis for thermal and mechanical allodynia accompanying *Staphylococcus aureus* infection (Tran et al. 2013). Also, N-formyl peptides (the metabolic by-products of all bacteria) stimulate G protein-coupled nociceptors (formyl peptide receptor 1) to elicit pain sensation (Tran et al. 2013). Viral and fungal pathogens are also not exempted from the direct-infection-induced pain. Although the mechanism behind nociceptive stimulation remains unclear, herpes simplex virus 1, herpes simplex virus 2 and varicella-zoster virus infections triggered varied pain sensations whilst fungal pathogen *Candida albicans* was also associated with pain thrush possibly caused by calcium influx into nociceptors (Nagel & Don 2014; Tong et al. 2010).

In human malarial infection, haemolysis of parasitised red blood cells releases parasites, their allied endotoxins (haemozoin and parasite deoxyribonucleic acid) and other cellular components that trigger Toll-like receptor 9 stimulation (Parroche et al. 2007) and tumour necrosis factor secretion (Wassmer et al. 2015). These physiological processes are known to activate the infection-inflammation-induced pain pathway (Miller et al. 2009; Pinho-ribeiro et al. 2017; Schedlowski et al. 2014), an observation corroborated by various clinical case reviews where malaria is documented to present with fever, chills, vomiting and various pains such as headache, myalgia, abdominal pain or back pain (Reisinger et al. 2005; Sarma & Kumar 1998).

Despite reported body pain in malaria patients, there is scanty information on malaria-related pain in animal model for malaria. Also, there is no report yet on contribution or association of malaria-related pain sensitivity to malaria-linked mortality. Although severe anaemia and cerebral malaria are known to be the typical pathological hallmarks that lead to death in untreated malaria infection, functional data from the study of pain are, however, shifting the aetiological causes of most mortalities to pain. Torrance and co-workers associated severe chronic pain to increase mortality irrespective of socio-demographic factors (Torrance et al. 2010).

This study was designed to investigate pain sensitivity in malarial infection with a view to improve our understanding on handling noxious sensation during malaria attack. Against our assumption, we observed that increased pain sensitivity reported in human was absent in animal-infected with malaria. In fact, pain sensitivity continued to decline as parasitaemia level increased in the Swiss mice used.

## Methods

### Animals and drugs

#### Mice and parasites

Orders for Swiss mice of the same progeny were placed at Central Animal Facility (CAF) in the University of Ilorin, Nigeria, several weeks before the commencement of these experiments. The same progeny set of animals was used to eliminate possible genetic bias. The mice used were 8- to 12-week-old male that had been subsequently backcrossed for at least five generations. Male mice were adopted to eliminate reported sex difference in pain sensation test, although this variation appeared to be absent in *Pb*-infected mice but was substantial in the control, intact group. The mice were moved from CAF where they were bred to resident in the College of Health Sciences Animals Facility, University of Ilorin, Nigeria, where they were allowed to habituate to experimental conditions and handling before these studies.

Animals were randomly divided into two groups of *Plasmodium berghei* (*Pb)*-infected and intact (uninfected) mice. The chloroquine-resistant *Pb* used was provided by the Malaria Research Laboratories, Institute for Advance Medical Research and Training, College of Medicine, University of Ibadan, Nigeria. Experimental mice were inoculated by intraperitoneal (*i.p.*) injection of 1 × 10^7^ parasitised erythrocytes from donor mice of 18% – 20% parasitaemia level. *Plasmodium berghei* strain was used because the model has gained wider scientific acceptance for its reproducibility and excellent mimicking of life cycle, structure, physiology and other crucial aspects of humans’ malarial parasites. It is one of the malarial parasites that infect mammals (murine rodents) other than humans. Percentage parasitaemia was assessed by estimating the percentage of parasitised-red blood cells in Giemsa-stained thin blood smears using a light microscope. Behavioural tests were carried out in the intact and infected mice within days 3 and 5 of the experiment.

At the end of the behavioural study, the animals were anaesthetised with ketamine hydrochloride (50 mg/kg; IM) and a thoracoabdominal incision was performed to expose the heart. One mL Nipro disposable syringe with 27-gauge needle was used for cardiac puncture and collection of blood. The blood was put in the heparinised Eppendorf tube and centrifuged for 15 min at 3000 rpm. The plasma was extracted into a plain Eppendorf tube, kept at -20 °C until use and was used to determine selected biochemical parameters. Following blood collection, the mice were transcardially perfused with 20 mL ice-cold phosphate-buffered saline (PBS) and the brains were quickly extracted, homogenised with 0.25 M sucrose solution and centrifuged at 3000 rpm for 15 min. The supernatant was aspirated into plain Eppendorf tubes and used to assay some brain-biochemical factors.

#### Drugs

Hydralazine hydrochloride (MKBS4863V), ketanserin (+)-tartrate salt (MKBR2179V), propranolol hydrochloride (BCBL8710V), phentolamine hydrochloride (085M4021V) and yohimbine hydrochloride (BCBM8231V) were procured from Sigma-Aldrich, St. Louis, Missouri, United States (US). The drugs were prepared in PBS prior to their *i.p.* administrations at doses of 5 mg/kg, 0.3 mg/kg, 2 mg/kg, 10 mg/kg and 10 mg/kg, respectively. The infected animals were observed until erythrocyte-stage of *Pb* was established (this coincided with day 3 post-inoculation) and treatments with the drugs commenced immediately. The treatment followed a 6-dose regimen of 0, 8, 24, 36, 48 and 60 h for both intact and *Pb-*infected mice. Thirty minutes after the last treatments on day 5, relevant behavioural and biochemical tests were conducted.

Naloxone hydrochloride (Samarth Life Sciences Pvt. Ltd., Mumbai, India (INLXB1602)) injection (400 *µ*g/mL) was administered via *i.p.* at a dose of 5 mg/kg whilst the controls were given 5 mL/kg of PBS. Treatments were carried out twice daily (12-h interval), starting on day 3 post-*i.p.* inoculation of *Pb.* Thirty minutes after last treatments on day 5, pain-related behavioural responses were evaluated in all animals.

### Relevance of the drugs to the study

Almost all the aforementioned drugs are antagonists meant to probe the contribution of precise receptors to malaria-related nociception and mortality. Briefly, hydralazine hydrochloride was used to inhibit monoamine reuptake whilst ketanserin (+)-tartrate salt, propranolol hydrochloride, phentolamine hydrochloride and yohimbine hydrochloride were, respectively, used to block serotonin receptor type 2, all beta adrenergic receptors, all alpha adrenergic receptors and alpha receptor type 2. Also, naloxone hydrochloride was used to block opioid receptors.

### Assessment of weight, blood glucose and haematological parameters

#### Body weight

The weight of each mouse was measured using a Camry digital scale (Camry, Zhongshan, China). The scale sensitivity is 0.1 gram and has a maximum capacity to measure 2 kg. The mice were measured by placing each mouse on the centre-top of the scale. To avoid unwanted movement, the mouse was put in a light-weight perforated carton, covered and placed on the scale. Prior to this, the carton was tared to zero. The display weight was recorded in gram (g).

#### Fasting blood glucose

Glucose level was assessed with an Accu-Chek Active glucometer (Roche Diabetes Care GmbH, Mannheim, Germany). Glucose level was assessed from a drop of tail vein blood on the Accu-Chek Active test strip inserted into the meter. Value was displayed in mg/dl.

#### Haematological parameters

This was performed using a Sysmex KX-21N Automated Haematology Analyer (Sysmex America, Inc., Mundelein, US). About 0.8 ± 0.3 mL of blood sample was collected via cardiac puncture from each mouse into the tri-potassium ethylenediamine tetra-acetic acid (K_3_EDTA)-coated sample bottle. The whole blood volume needed is 50 *µ*L, and detection principle for blood elements is the direct current detection method with automatic discriminators that separate the cell populations based on complex algorithms. For haemoglobin and haematocrit, the detection principles are non-cyanide method and cumulative pulse height detection method, respectively. Sysmex cell counters have an exceeding uncompromised precision and accuracy regardless of the samples’ quantities or concentrations.

#### Kits

Serotonin and noradrenaline enzyme-linked immunosorbent assay (ELISA) kits were from Elabscience Biotechnology Co., Ltd. Wuhan, China, and were used to evaluate the level of these neurotransmitters in the plasma and brain. Briefly, 50 *µ*L of sample or standard was added to a microtiter plate that had been pre-coated separately with serotonin and noradrenaline. 50 *µ*L of biotinylated detection antibody (Ab) specific for either serotonin or noradrenaline was added to each well. The microtiter plate with its contents was incubated for 45 min at 37 °C. At the expiration of incubation time, excess conjugate and unbounded sample or standard were washed from the plate. This was immediately followed by addition of 100 *µ*L of Horseradish Peroxidase to each microplate well and then incubated for 30 min at 37 °C. The incubation was followed by washing of the plate and addition of 90 *µ*L of TMB substrate solution to each well. The enzyme-substrate reaction was allowed for 15 min and was stopped with sulphuric acid solution. The change in colour was quantified using a plate reader at a wavelength of 450 nm. The concentration of serotonin and noradrenaline was determined by comparing the absorbance to the standard curves.

#### Enzymes

Protein was measured as described by Lowry et al. (1951); (Lowry et al. 1951) using bovine serum albumin as the standard. Activities of catalase, superoxide dismutase and reduced glutathione were evaluated using methods described by Beers and Sizer (1952) (Beers & Sizer 1952), Misra and Fridovich (1972) (Misra & Fridovich 1972) and Jollow et al. (1974) (Jollow et al. 1974), respectively. However, activities of Ca^2+^ – Mg^2+^ ATPase and Na^+^ – K^+^ ATPase were assessed using Graham and Jack (1990) and Jarrett and Penniston (1977) methods, respectively (Graham & Jack 1990; Jarrett & Penniston 1977).

### Evaluation of sensory pain sensation

#### Formalin pain test

Pain and its related behavioural response were evaluated using formalin test (Hunskaar & Hole 1987). Briefly, 20 *µ*L of 2.5% formalin in PBS was injected into the plantar surface of the right hind paw. Each mouse was placed in a round transparent plastic (20 cm in diameter) or Open Field Maze and videotaped for 50 min post-injection of formalin. The total time spent licking the injected hind paw (behavioural response to pain) was documented and used to quantify pain perception. In this model, increased pain behavioural response (paw licking, shaking and lifting) means increase in pain sensitivity or perception and vice versa. This interpretation is symmetrical to the one in the literature and its history of ethological validity remains consistent for decades (Hunskaar, Fasmer & Hole 1985; Tabata-Imai, Inoue & Mori 2014; Yin et al. 2016). This formalin pain model is preferred as it needed only one stimulus to elicit both neurogenic and inflammatory pain. The first 5 min after injection of formalin was taken as early phase (neurogenic pain) whilst the period between 20 min and 50 min post-injection was recorded as late phase (inflammatory pain). Data were extracted in quadruplicate by trained observers blinded to the experimental design, and average values were calculated.

#### Foot stamp test

Immediately after the formalin test, the two hind limbs (formalin-injected and intact paw) were dipped into yellow and blue ink-soaked sponges, respectively. The mice were permitted to walk individually on a white paper placed in their respective resident cage for 2 min (Nagae, Hiraga & Yoneda 2007). Pain perception in mice was reported as % usage of formalin-injected foot, as shown below.

$$\begin{array}{l} Pain\;perception=\\ \frac{\text{Number of Formalin-Injected Foot Stamps }(\text{Red colour})}{\text{Number of in act foot Stamps }(\text{Black colour})}\times 100 \end{array}$$

**Von Frey test:** Mechanical sensitivity was evaluated by measuring the threshold required for the withdrawal of the right hind paw (Lambert, Mallos & Zagami 2009) against von Frey filament. Summarily, the ventral surface of hind paw was stabbed with the filament using feeble but enough pressure to cause bucking. The filament was removed after 5 s for any unresponsive mouse. For responsive animals, the value (calibration) on von Frey filament was accepted as withdrawal threshold if the hind paw is withdrawn at minimum of five times from seven stimuli applied.

**Cold plate test**: Each mouse was put in a perforated restriction glass chamber arranged on a thin glass surface platform. The restriction chambers were separated with blinds to prevent the animals from seeing each other. After acclimatisation, ice-pellet was applied on the right hind paw until the mouse exhibited pain-related behaviours such as lifting or moving away the affected limb. Time taken to display any of these behaviours is taken as the end-point. The procedure was repeated three times with 2-min interval, and final mean value was documented for each mouse (Kohan et al. 2002).

### Assessment of motor function and pathological changes in the brain

#### Open field maze

The motor function of the mice was assessed using a (40 cm × 40 cm × 40 cm) tiled floor 16 square cells open field maze. It was first described in 1932 and popularly used in the evaluation of anxiety in rodent. Open field test can be adapted to assess nonanxiogenic phenomena such as memory, exploration and locomotor activity (Deacon 2006). Mouse was introduced to the centre square cell of the maze with the mouse not facing the handler. Each mouse explored the novel environment for 5 min, and all activities displaced in the maze were captured by a mounted video camera just above the paradigm. As described by Seibenhener and Wooten (2015), the following indices for motor function were extracted by four persons blinded to the experiment and, average values of indices: ambulatory activity, rearing-time and frequency were recorded. Similar or increased horizontal (ambulation) and vertical (rearing) locomotive behaviour with intact animals suggest motor competence (Bichler et al. 2013; Choi et al. 2017).

#### Assessment of pathological changes in the frontal cortex

After pain test on day 4, mice were anaesthetised, perfused successively with cold PBS and 4% PBS-paraformaldehyde via cardiac puncture. Brains were then removed, immersed in 4% PBS-paraformaldehyde and kept for 24 h at 4 °C. Thereafter, tissues were processed using ascending grades of ethanol, xylene (for clearing) and paraffin wax (for embedding). Frontal cortex was sectioned (5*µ*m; MK 1110 rotary microtome) and stained with Cresyl fast violet (CFV) and haematoxylin and eosin (H&E). Also, immunohistochemistry was carried out on the sections of frontal cortex using anti-mouse-CD68 (1:100; BIO-RAD, United Kingdom). Basically, sectioned tissues were deparaffinised, underwent antigen retrieval and subjected to CD68 antibody interaction. Photomicrographs of varied stained tissues were captured using the Zeiss Axiostar plus light microscope.

### Statistical analysis

Unpaired *t*-tests, one-way ANOVA with Bonferroni’s *post hoc* test and Log-rank (Kaplan-Meier) test were used. IBM SPSS Statistics (software version 24, Armonk, New York, US) and Prism (GraphPad Software version 7.0, La Jolla, California, US) were used for analyses.

### Ethical consideration

All procedures used were in accordance with the approval from the University Ethical Review Committee (Approval Number: UERC/ASN/2016/485), University of Ilorin, Ilorin, Nigeria. These techniques were in harmony with relevant institutional guidelines and regulations for Animal Experiment and Welfare. Also, these guidelines are in strict compliance with the applicable section of National Institutes of Health guides (NIH Publications No. 8023, revised 1978) for the care and use of Laboratory animals.

## Results

Data of interest were assessed (at Day 4 post-*Pb* inoculation except otherwise stated) in quadruplicate, and the averages were accepted as value for each parameter. Results as presented were from one experiment representative of two experiments performed.

### *Pb-*induced malaria causes alteration in Body weight, fasting blood glucose and haematological parameters in Swiss mice

Intact animals gained significant weight when their initial weights were compared with their final weights. For *Pb-*infected mice, there was a decrease in the final body weight compared to initial weights (Figure 1a), although this was not significantly different. However, when weight difference was compared, there was a significant decrease in the body weight of *Pb-*infected mice compared to intact mice (Figure 1b). Also, the fasting blood glucose level on Day 5 significantly decreased in *Pb-*infected mice when compared to intact mice (Figure 1c).

**FIGURE 1:**
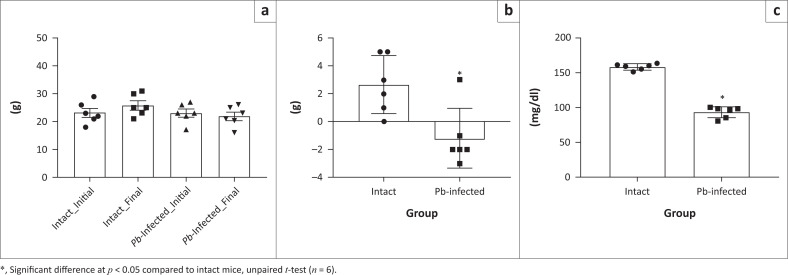
Body weight and blood glucose following *Pb* infection in Swiss mice. (a) Final body weight compared with initial weight. (b) Body weight difference pre- and post-infection periods. (c) Fasting blood glucose level at Day 5.

**FIGURE 2:**
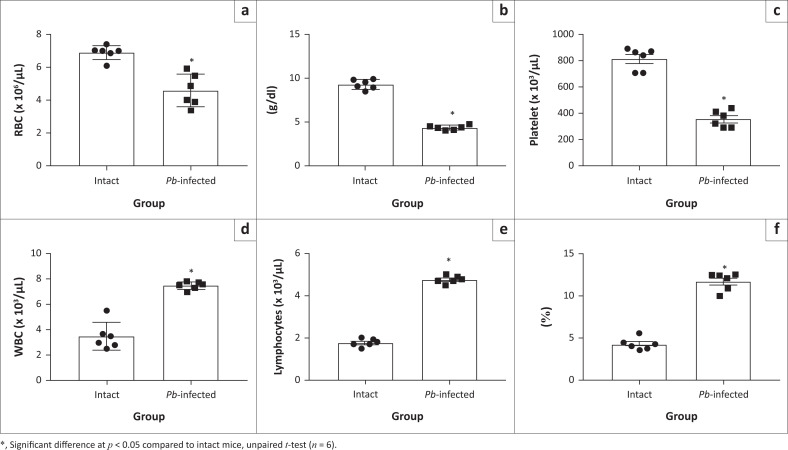
The haematological Parameters of *Pb-*infected and intact (uninfected) mice. (a) Red blood cells count. (b) Haemoglobin level. (c) Platelets count. (d) White blood cells count. (e) Lymphocytes count. (f) Platelet large cell ratio.

Similarly, red blood cell count (Figure 2a), haemoglobin level (Figure 2b) and platelet cell count (Figure 2c) decreased significantly in *Pb*-infected mice compared to intact mice. However, the white blood cell count (Figure 2d), lymphocytes count (Figure 2e) and platelet large cell ratio (Figure 2f) were significantly increased in *Pb*-infected mice compared to intact mice.

### *Pb-*infection attenuates pain perception

**FIGURE 3:**
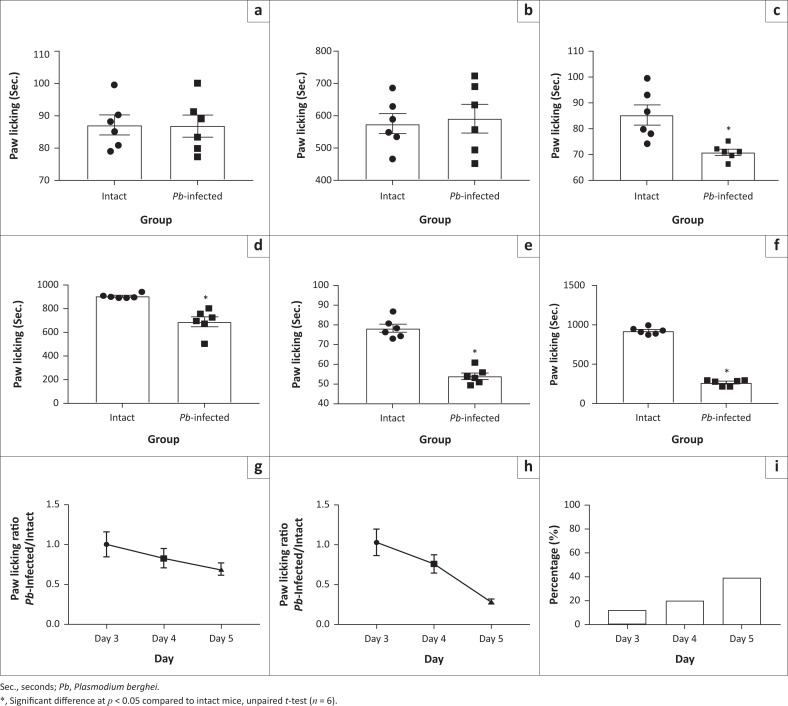
*Pb*-induced analgesia in mice. (a) and (b) Time spent on paw licking during the early (0–5 min) and late (20–50 min) phase of formalin test on Day 3 post-*Pb* inoculation (early phase and late phase), respectively. (c) and (d) Time spent on paw licking during the early (0–5 min) and late (20–50 min) phase of formalin test on Day 4 post-*Pb* inoculation (early phase and late phase), respectively. (e) and (f) Time spent on paw licking during the early (0–5 min) and late (20–50 min) phase of formalin test on Day 5 post-*Pb* inoculation (early phase and late phase), respectively. (g) and (h) Successive trend of pain sensitivity during the neurogenic and inflammatory pain (early phase pain perception and late phase pain perception), respectively. (i) Percentage parasitaemia level on Days 3, 4 and 5.

Pain as one of the common symptoms of malaria in human was probed in an animal model for malaria. On day 3 post-parasite inoculation, pain sensitivity was not statistically different in the infected mice compared to intact mice (Figures 3a and b). On Day 4, however, there was a significant decrease in sensitivity to neurogenic and inflammatory pain in the *Pb*-infected mice compared to intact animals (Figures 3c and d). This decrease in pain sensitivity was more striking on Day 5 probably because the percentage parasitaemia was becoming more life-threatening. Precisely, there was a significant decrease in pain perception in *Pb*-infected animals compared to intact mice (Figures 3e and f) during both early and late phase of formalin test. To investigate the trend of pain perception within the 3 days of purview, the ratio of behavioural responses to formalin pain in *Pb*-infected and intact mice was plotted and it showed a decline in perception curves for neurogenic and inflammatory pain (Figures 3g and h). The percentages of parasitaemia within these days were 12.54% (Day 3), 20.35% (Day 4) and 39.11% (Day 5); (Figure 3i).

**FIGURE 4:**
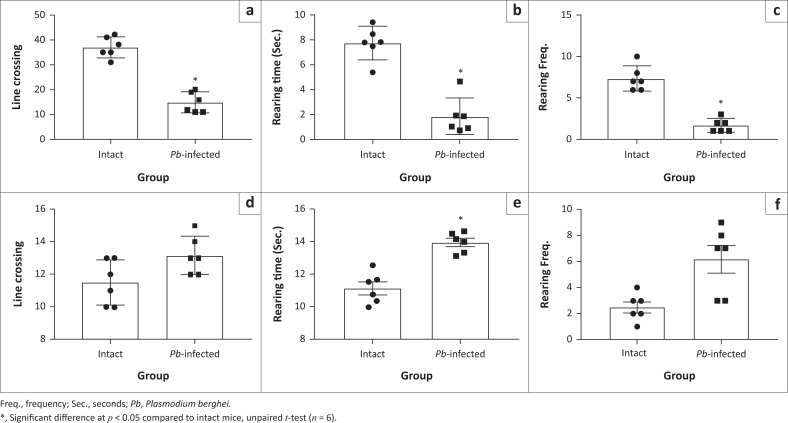
Motor behavioural indices in *Pb-*induced malaria. (a) Number of line crossing during early phase of formalin test. (b) and (c) Time spent and frequency of rearing during the neurogenic pain phase of formalin test, respectively. (d) Number of line crossing during late (20–50 min) phase of formalin test. (e) and (f) Time spent and frequency of rearing during the inflammatory pain (late) phase of formalin test, respectively.

**FIGURE 5:**
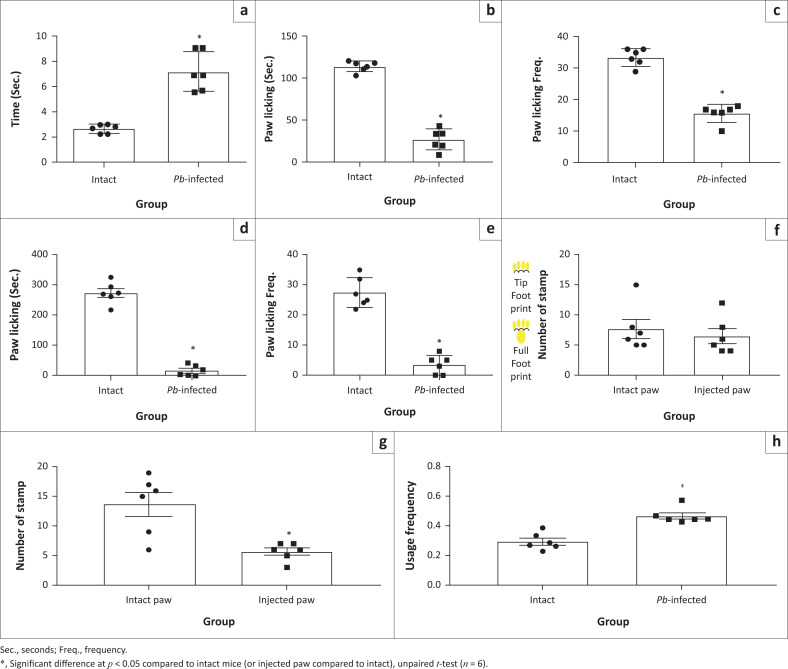
*Pb-*induced malaria ameliorates chronic pain perception. (a) ‘Latent period’: Time taken to make the first paw lick behaviour after formalin injection. (b) Early phase paw-licking (0–5 min) time during formalin test. (c) Frequency of paw licking during the early phase. (d) Late phase paw licking (20–50 min) duration during formalin test. (e) Frequency of paw licking during the late phase. (f) Number of full footprint of injected paws in *Pb*-infected mice. (g) Number of full footprint of injected paws in intact mice. (h) Tolerability for using injected paw to intact paw.

To ascertain that the observed analgesic-like effect recorded in *Pb-*infected mice was not because of compromised motor function, motor activities were simultaneously assessed with formalin pain test on open field maze at Day 4 post-*Pb* inoculation. Although ambulation (horizontal movement; Figure 4a) and rearing behaviours (vertical movement; Figures 4b and c) were significantly decreased in *Pb*-infected mice compared to intact mice during the early phase of formalin test (0–5 min), these behaviours improved better at the late pain phase (20–50 min), showing that the motor function in the infected mice (Figures 4d, e and f) was relatively viable. Also, a significant (*p* < 0.05) increase in ‘latent period’ (duration between injection of formalin and the first paw-licking behaviour) was observed in the *Pb-*infected animals compared to the intact ones (Figure 5a). Again, both early (Figures 5b and c) and late (Figures 5d and e) pain-related behaviours (paw licking time and frequency) decreased significantly in *Pb*-infected animals compared to intact mice, corroborating the earlier observations. To affirm the observed ameliorated pain perception, foot stamp test was carried out. When the plantar surface of the hind paw is injected with formalin solution (1% – 5%), a mouse with normal sensory perception uses more of its foot-tip than the full footprint (Figure 5f). The full footprint count for the injected limb compared to the intact limb was not significantly different in the *Pb*-infected mice (Figure 5f). In the intact mice, there was a significant decrease in the full footprint counts of the injected foot (Figure 5g) compared to intact foot. Between the two groups however, *Pb*-infected mice exhibited a significant increase in full-foot (injected paw/intact paw) count compared to the intact mice (Figure 5h). Lastly, we attempted to investigate whether decreased pain sensitivity was limited to noxious chemical only, and von Frey test and cold-plate test were carried out. The results also showed a significant decrease in pain perception to noxious mechanical and thermal stimuli in *Pb-*infected mice compared to the intact animals (Appendix 1, Figures 1-A1[a] and 1-A1[b]).

**FIGURE 6:**
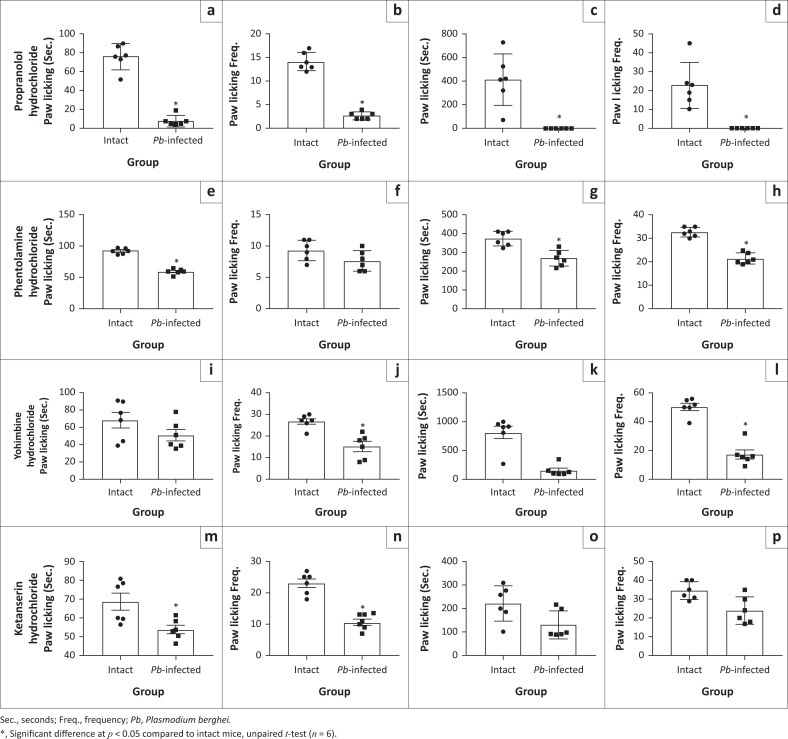
Role of adrenergic and serotoninergic system in *Pb*-induced analgesia. (a) and (b) Time spent and frequency of paw licking at the early phase (0–5 min) of formalin test in propranolol hydrochloride-treated mice, respectively. (c) and (d) Time spent and frequency of paw licking during the late phase (20–50 min) of formalin test in propranolol hydrochloride-treated mice, respectively. (e) and (f) Time spent and frequency of paw licking at the early phase (0–5 min) of formalin test in phentolamine hydrochloride-treated mice, respectively. (g) and (h) Time spent and frequency of paw licking at the late phase (20–50 min) of formalin test in phentolamine hydrochloride-treated mice, respectively. (i) and (j) Time spent and frequency of paw licking during the early phase (0–5 min) of formalin test in yohimbine hydrochloride-treated mice, respectively. (k) and (l) Time spent and frequency of paw licking during the late phase (20–50 min) of formalin test in yohimbine hydrochloride-treated mice, respectively. (m) and (n) Time spent and frequency of paw licking at the early phase (0–5 min) of formalin test in ketanserin hydrochloride-treated mice, respectively. (o) and (p) Time spent and frequency of paw licking during the late phase (20–50 min) of formalin test in ketanserin hydrochloride-treated mice, respectively.

**FIGURE 7:**
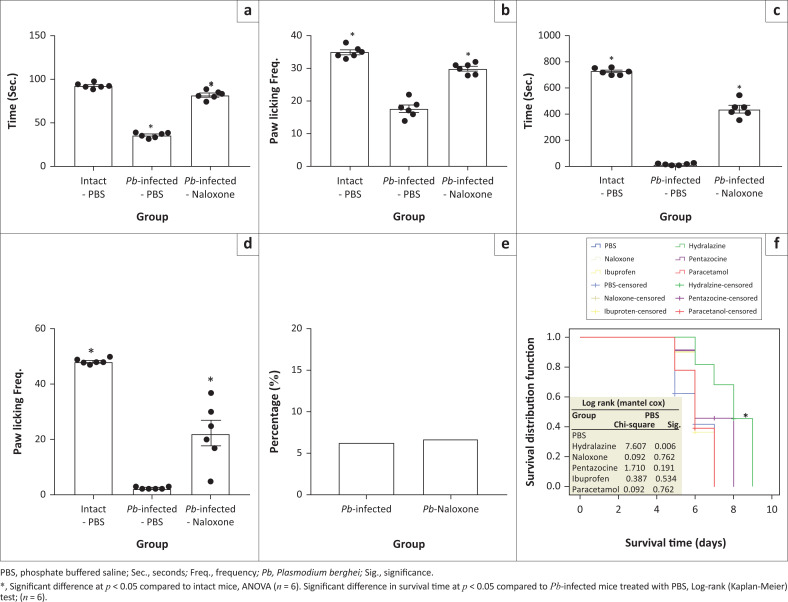
Impact of naloxone on pain sensitivity and survival time in *Pb-*infected mice. (a) and (b) Time used for paw licking and its frequency during the early phase (0–5 min) of formalin test, respectively. (c) and (d) Time spent on paw licking and its frequency during the late phase (20–50 min) of formalin test. (e) Percentage parasitaemia level. (f) Survival time in *Pb*-infected mice.

When propranolol and phentolamine hydrochloride were administered to probe adrenergic involvement, similar pattern of diminished pain sensitivity was exhibited during early and late phase of formalin test in the *Pb-*infected mice compared to intact mice (Figures 6a–h). Also, treatment with yohimbine hydrochloride (α_2_ – antagonist) demonstrated similar decrease in pain-related behaviours except that paw licking time at the early phase was not significantly different in *Pb*-infected mice compared to intact animals (Figures 6i–l). Serotoninergic probing with ketanserin hydrochloride (5-HT2AR antagonist) showed similar significant decrease in early phase pain behaviour in the *Pb-*infected mice (Figures 6m and n) compared to intact animals but pain sensitivity at the late phase was not significantly different in *Pb*-infected mice compared to intact animals (Figures 6o and p). However, the analgesic-like effect in *Pb-*infected mice was blocked following naloxone treatment (Figures 7a–d). This made us to assume that naloxone probably had anti-malarial effect. Our investigation of this assumption showed almost no difference in percentage parasitaemia between *Pb-*infected mice and naloxone-treated animals (Figure 7e).

### Pain has little or no contribution to malaria-related death

To investigate contribution of pain system in malaria-related mortality, various drugs known to interfere with pain processing system were used to investigate animals’ survival time. There was no significant difference in the survival time between animals treated with acetaminophen, pentazocine, ibuprofen, phentolamine and naloxone compared with placebo (PBS)-treated *Pb*-infected group. However, about 20% of hydralazine-treated animals survived till Day 9, about 48 h after the death of animals in other groups (Figure 7f).

### *Pb*-induced malaria elicits changes in selected ATP-hydrolytic enzymes, neurotransmitters and oxidative enzymes in Swiss mice

**FIGURE 8:**
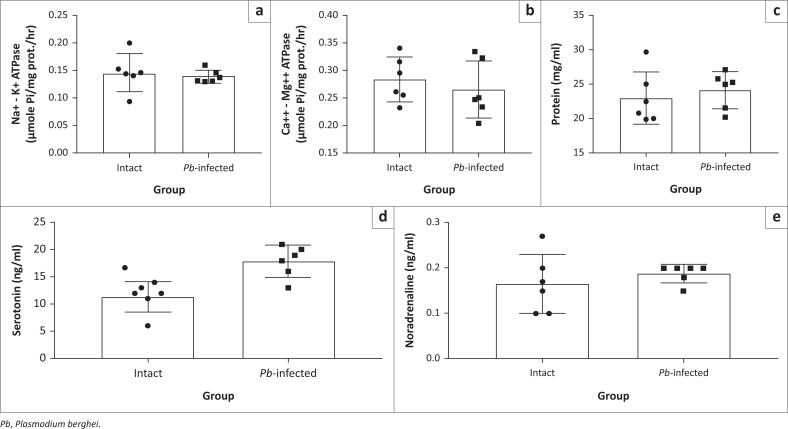
Biochemical changes in the brain of *Pb*-infected Swiss mice. (a) and (b) Brain activities of Na^+^ – K^+^ ATPase and Ca^2+^ – Mg^2+^ ATPase, respectively. (c) Total brain protein level. (d) and (e) Levels of serotonin and noradrenaline in the brain, respectively.

**FIGURE 9:**
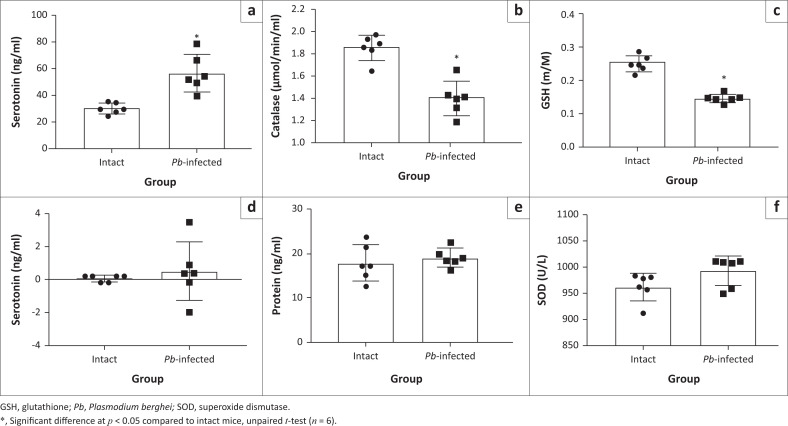
Changes in neurotransmitter and antioxidant enzymes in the plasma of *Pb*-infected Swiss mice. (a) Plasma serotonin level. (b) and (c) Activities of catalase and glutathione peroxidase, respectively. (d) Plasma noradrenaline level. (e) Total plasma protein level. (f) Superoxide dismutase activity.

We then investigated enzymes necessary for the utilisation of metabolic energy in the brain of the experimental mice. Na-K ATPase and Ca-Mg ATPase were not significantly altered in Pb-infected mice compared to that of intact mice (Figures 8a and b). The protein level was not different either (Figure 8c) but there was a biological increase in the brain serotonin level for the *Pb-*infected mice compared to intact mice (Figure 8d); this, however, is not statistically different. Also, the noradrenaline level in the *Pb*-infected mice was not significantly different compared with the intact mice (Figure 8e). In the plasma, serotonin level in the *Pb*-infected mice increased significantly compared with the intact animals (Figure 9a). Also, catalase and glutathione peroxidase activities decreased significantly in *Pb*-infected mice compared to intact mice (Figures 9b and c). Plasma noradrenaline, protein level and superoxide dismutase activity in *Pb*-infected mice were not significantly different compared with the intact mice (Figures 9d, e and f).

### *Pb* induces slight morphological change in the brain

To exclude the possibility that experimentally induced cerebral malaria was responsible for the observed difference in pain sensitivity on Day 4, sections of the brains were surveyed for activated microglia using antibody to macrophage marker CD68. Following injury or alterations in their parenchyma microenvironment, resident microglia in healthy brain are rapidly activated and assumed typical morphological thickening with rounded amoeboid shape. Areas involved in pain, fear (cingulate cortices) and locomotor (primary motor cortex) were primarily evaluated.

**FIGURE 10:**
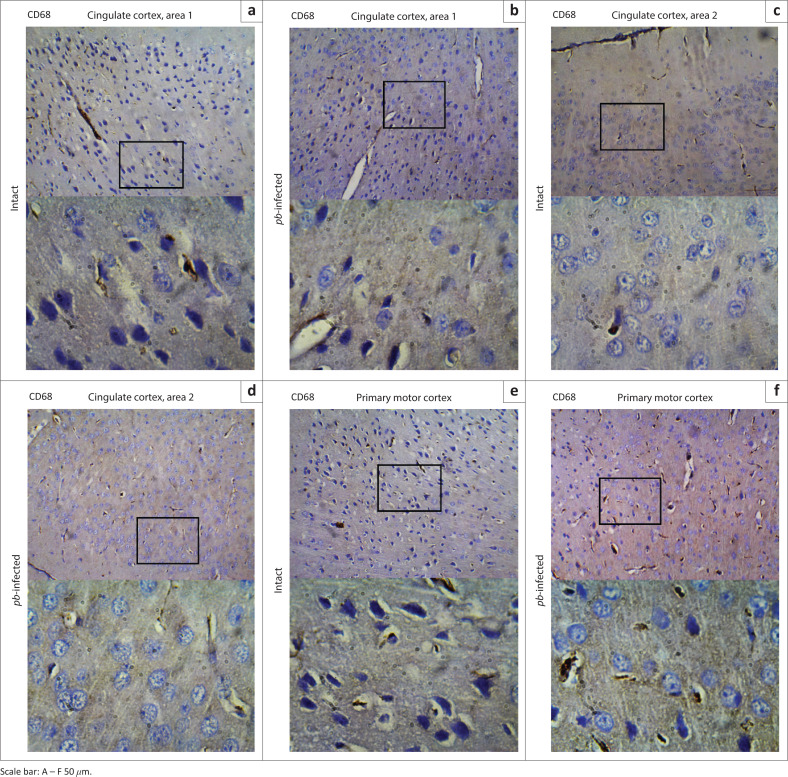
Microglial immunostaining within the frontal part of the cerebral cortex. (a) and (b) Anti-CD68 immunostaining sections of cingulate cortex area 1 in intact and *Pb*-infected mice, respectively. (c) and (d) Staining cingulate cortex area 2 with Anti-CD68 antibody in intact and *Pb*-Infected mice, respectively. (e) and (f) Respective immunostaining of the primary motor area with Anti-CD68 antibody in intact and *Pb*-infected mice. There was no immunopositive cell in all the parts of the brain investigated with the marker for cerebral malaria.

**FIGURE 11:**
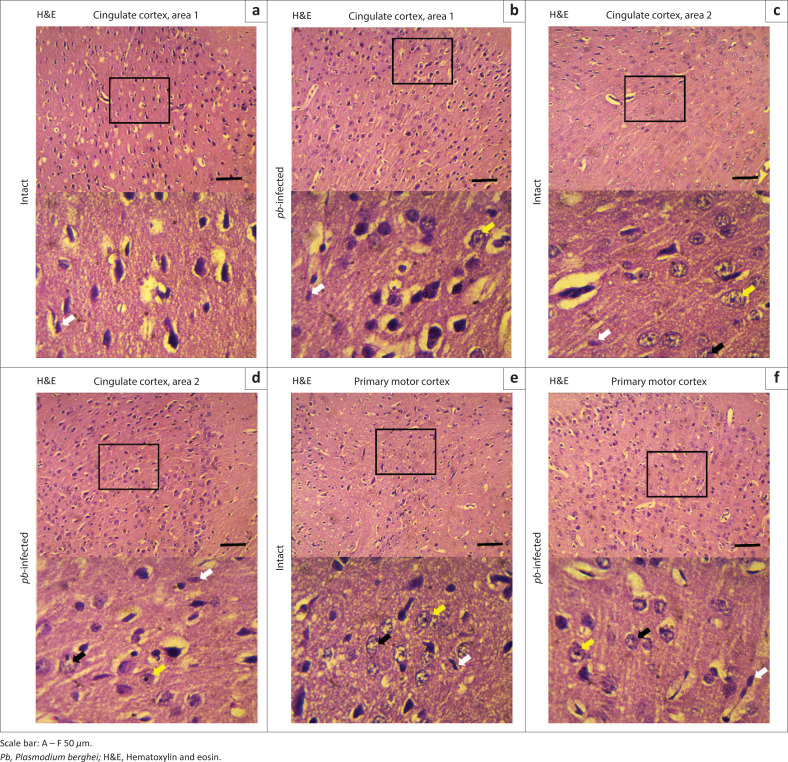
Neuronal histochemistry of cerebral cortex. (a) and (b) Haematoxylin and eosin-stained sections of cingulate cortex area 1 in intact and *Pb*-infected mice, respectively. (c) and (d) Neural cells distribution in cingulate cortex area 2 in intact and *Pb*-Infected mice, respectively. (e) and (f) Morphology of neural cells at the primary motor area of intact and *Pb*-infected mice in Haematoxylin and eosin-stained sections, respectively. Despite both *Pb*-infected and intact mice appeared to have symmetrical cytoarchitecture and cell distribution, several nerve cells appeared fading with centralised nuclei (yellow arrow). There are also few neural cells that appeared ghost-like (black arrow) with peripheral nuclei, yet most of the microglia present (white arrow) are not activated.

**FIGURE 12:**
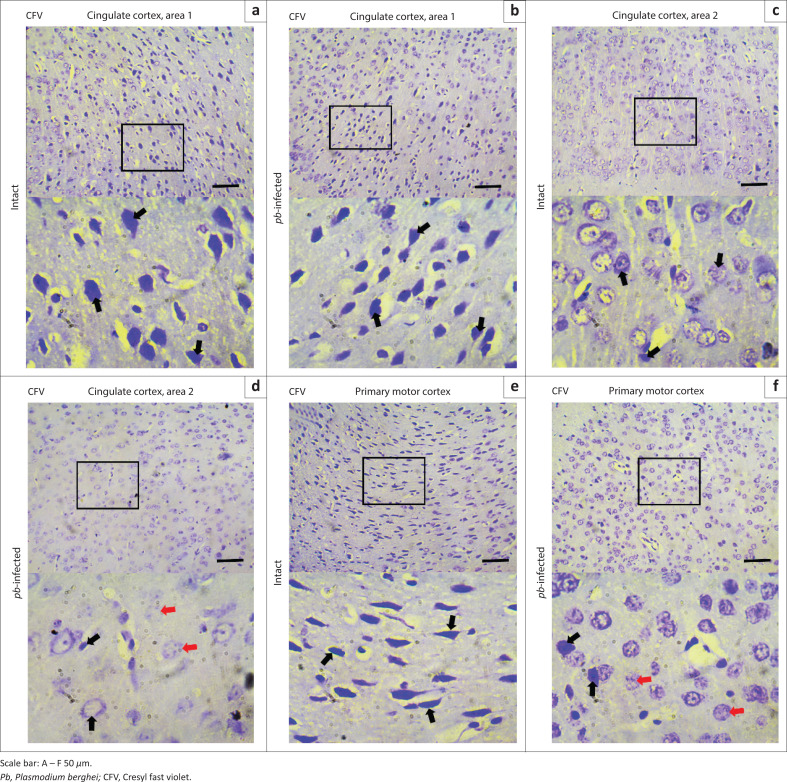
Neuronal population within the midline of brain. (a) and (b) Cresyl violet-stained sections of cingulate cortex area 1 in intact and *Pb*-infected mice, respectively. (c) and (d) Nissl substance distribution in Cresyl violet-stained sections of cingulate cortex area 2 in intact and *Pb*-infected mice, respectively. (e) and (f) Primary motor cortex in intact and *Pb*-infected mice stained with Cresyl violet, respectively. Note, almost all the cell bodies stained remarkably (black arrow) although few of them appeared pale and assumed round-shaped (red arrow).

In each three representative mouse, CD68 revealing macrophages were undetectable either along the borders of four serially sectioned frontal cortices or within the core areas of cingulate cortices (Figures 10a–d) and primary motor cortex (Figures 10e–f). Absence of this transmembrane glycoprotein suggested that both intact and *Pb*-infected mice enjoyed a relatively quiescent microenvironment. Cell morphology was also examined using haematoxylin and eosin-stained sections. Cingulate cortices (Figures 11a–d) and primary motor cortex (Figures 11e–f) stained intensely and showed a mixture of small- to medium-sized neurons. The neuronal cells appeared to have symmetrical cell distribution and cytoarchitecture. Sections from intact mice exhibited copious intact cells with centralised nuclei (yellow arrow) in contrast to sections from infected mice where fewer cells appeared ghost-like (black arrow) with nuclei pushed to the periphery. All tissues (Figures 11a–f) showed fewer resting microglia (white arrow). Nuclei of resting microglia are recognised with their dark stain, distinguished rod-, elongated- or ‘cigar’-shaped and usually irregularly contoured. Cresyl fast violet-stained sections showed prominent cell bodies (black arrow) that signify abundant ribosomes attached on rough endoplasmic reticulum in the cingulate cortices (Figures 12a–d) of intact and *Pb-*infected animals, although Nissl substance in cingulate cortex area 2 of *Pb-*infected mice (Figure 12d) appeared pale (red arrow). Also, primary motor area in the intact animals (Figure 12e) showed copious cell bodies (black arrow) compared to the *Pb*-infected (Figure 12f) mice where fewer pyramidal cell bodies appeared faintly stained and assumed round-shaped (red arrow).

## Discussion

The present study established *Pb*-induced analgesic-like effect in an animal model for malaria during life-threatening stage. This analgesic-like effect was enhanced with daily increase in parasitaemia level, and it was shown by a striking diminished pain-related behaviours. The effect seems to have central synergistic contributions of α_2_, µ-opioid and 5HT2A receptors. This assertion was drawn from the facts that when antagonists of α_2_, µ-opioid and 5HT2A receptor were used, there was increase in pain-related behaviours (that is, improved in pain sensitivity) in the *Pb*-infected mice during the neurogenic (early) and or inflammatory (late) phase of formalin pain test compared to intact animals. Precisely, nociceptive behaviour in *Pb*-infected mice was improved with naloxone (µ-opioid antagonist; both early and late phases), yohimbine hydrochloride (α_2_ antagonist; early phase) and ketanserin hydrochloride (5HT2A antagonist; late phase). Also, results from this study showed little or no contribution of the pain processing system on malaria-related death in infected mice. Survival time was similar to mice treated with drugs capable of dampening or enhancing pain perception. This study is important as it captures the neglected spectrum of neural physiology that precedes experimental cerebral malaria and death in animal model for malaria. Despite accumulated evidence that showed close similarity between animal models for malaria and malaria presentation in humans, the present findings showed that neural handling of sensory (pain) sensation in this animal model contrasts the one obtainable in humans. Also, malaria infection abolished reported sex-difference responses to noxious stimuli in mice.

Evidence-based studies demonstrated that parasitic infection can both increase and decrease pain perception (Huang et al. 2016; Kavaliers & Colwell 1993). Rodent-specific malarial parasite, *Plasmodium berghei*, consistently showed a decreased response to neurogenic and inflammatory pain with daily proliferation in mice. This contrasts obtainable claims in human studies where malaria-linked pain was widely reported (Sarma & Kumar 1998; Reisinger et al. 2005; Zaki 2010), and *Pb* parasites or their products at a critical stage mimicked analgesic effects in mice. The observed reduction in response to pain sensation seems to be associated with parasitaemia level. Infectious parasite-induced analgesic-like effect was also reported in *Eimeria vermiformis* and *Schistosoma mansoni* infection (Fiore et al. 1996; Kavaliers & Colwell 1993, 1994). The critical point when parasitic anti-nociception begins in this animal model for malarial infection seems to fall between > 12.54% and ≤ 20.35% parasitaemia level. This was drawn from this study based on the fact that on Day 3 post-*Pb* infection (at 12.54% parasitaemia level), pain-related behavioural responses to noxious formalin were not significantly different in *Pb*-infected mice compared to the intact mice. But 24 h later (Day 4, at 20.35% parasitaemia level), response to noxious chemical (2.5% formalin) decreased in *Pb*-infected mice relative to the intact mice. This critical point (> 12.54% and ≤ 20.35% parasitaemia level) was limited by inability to establish the specific time and parasitaemia level at which the decline in pain sensitivity commenced.

Notwithstanding, the critical period was hypothesised to coincide with experimental cerebral malaria in infected animals. The hypothesis was tested via investigating the presence of CD68 immunopositive cells in the brain sections of *Pb*-infected mice and also by examining other morphological alterations in H&E- and CFV-stained sections. CD68 revealing activated macrophages was unexpressed either in intact or *Pb*-infected mice. Resting microglia were noted in all H&E-stained sections (intact and *Pb*-infected), and there was no remarkable morphological or cytoarchitecture variation that could be linked to experimental cerebral malaria. Although there was slight dispersed Nissl substance in the cingulate cortex area 2 and primary motor cortex of *Pb*-infected mice, the stained cell bodies still showed the presence of sufficient amounts of Nissl body.

For the present study, pain-related behaviours are defined as different measurable series of well-coordinated, repetitive and reproducible muscular exertions displayed in response to noxious stimuli. Hence, these muscular exertions-linked behaviours can be affected in animals with compromised motor function. To ascertain viability of motor function in the infected mice, pain test was carried out in a novel open field arena. The maze has been reported to be useful in mood and motor function test (Albert & Istvan 2014). Our finding is in conflict with few claims in the literature where increased locomotion was associated with suppressed pain perception (i.e. decreased pain sensitivity) (Carstens & Moberg 2000; Sheahan et al. 2017). Specifically, despite increase in pain sensitivity (suppressed pain) in *Pb-*infected mice, early phase vertical (rearing) and horizontal (ambulation) locomotor activities were significantly decreased compared to the intact animals. However, during the late phase (20–50 min) of the formalin test, the intact animals spent much of their time on paw licking as against the *Pb*-infected mice; thus, the horizontal and vertical locomotor activities in the *Pb*-infected animals improved and were not significantly different compared to the intact mice. Together, these results showed that the motor function in the *Pb*-infected mice was still viable but might not be at its optimal performance capacity. Also, histological evaluation of primary motor area showed minor changes that could not be described as dysfunctional morphological alterations. These findings reinforce the claim for partial competence of the motor command generating area in the *Pb*-infected mice. Again, decreased pain sensitivity to noxious chemical (formalin) was re-demonstrated in mice and similar declined pain sensitivity was established in mechanical and thermal pain test (see Appendix 1, Figures 1-A1[a] and 1-A1[b]). The malaria-induced analgesic-like effect was, however, abolished in naloxone-treated mice without beneficial effect on the percentage parasitaemia level, thus showing possible involvement of opioidergic receptor. Naloxone blocks opioid receptors (Chen, Chen & Mao 2014), and it enhances response to pain when administered at high doses in animals (Mena, Mathur & Nayar 1996) or human beings (Levine, Gordon & Field 1979). Despite compelling reports on the roles of adrenergic system in pain modulation via its α and β receptors (Carroll, Mackey & Gaeta 2007; Takashi et al. 2017), antagonists of these receptors could not reverse the observed *Pb*-linked analgesia. Instead, the nonselective beta-receptor antagonist seemed to have further ‘hyperpolarised’ the pain system by almost eliminating early phase response to pain stimulus in infected mice and completely abolished pain-related behaviours during the late phase of formalin test. To the best of our knowledge, we could not find similar report in humans. It will be good to investigate whether people managing their high blood pressure with propranolol do experience a remarkable decrease in pain sensitivity during malaria attack. However, propranolol had been shown to decrease pain via its actions on β receptors in humans and rodents (Exposto et al. 2015; Petersen et al. 2018; Schweinhardt et al. 2013). Unlike the action of propranolol on β receptors, the alpha (α) receptors component of adrenergic system showed minimal or no effect on the observed analgesia when phentolamine hydrochloride (nonselective α-receptor antagonist) and yohimbine hydrochloride (α_2_-receptor antagonist) were used. The action of α_2_-receptor blocker in the amelioration of pain sensation as previously reported in animal studies (Pertovaara 1993; Takashi et al. 2017) was recorded in *Pb-*infected mice at the early phase of formalin test but not during the late phase probably because of the short half-life of the antagonist. Also, *Pb*-induced analgesia became perturbed at the late phase when 5-HT2A receptor antagonist was used, suggesting that α_2_- and 5-HT2A-receptor might contribute to the anti-nociceptive-like effects. Why 5-HT2A receptor antagonist used could not elicit similar effect during the early phase of formalin test is not known. However, action of the 5-HT2A receptor in pain modulation remains controversial. Compelling evidence showed that the 5-HT2A receptor could enhance (Abbott, Hong & Blier 1996; Cervantes-Duran, Rocha-Gonzalez & Granados-Soto 2013) or ameliorate (Honda et al. 2006; Li et al. 2013) pain perception in humans and rodents. It is, therefore, possible that synergistic effects of *Pb* parasites and/or their products on opioidergic, adrenergic and serotoninergic receptors in the host led to increased non-responsiveness to noxious chemical during a critical stage of *Pb* infection in mice.

The present study also showed a result on delayed perception of pain in the infected animals upon injection of formalin, a sharp contrast to what was observed in intact mice. Profound suppression of pain sensation to its minimum level without major alteration of the motor function demonstrated that only neural circuitry for pain seemed to be affected by the parasites and/or their products. The possibility of affected circuitry contributing to malaria-linked mortality cannot be overlooked. Investigating this, our results showed that there was no significant difference in the survival time for the infected animals treated with drugs capable of causing alteration in pain sensitivity or perception compared to placebo-treated mice. However, the monoamine-reuptake inhibitor (hydralazine hydrochloride) appeared to increase survival time in the hydralazine-treated mice compared to the placebo group. The survival of about 20% of these mice for extra hours could not be described as remarkable, and data were not sufficient to extrapolate any possible usefulness in preventing malaria-related death. Despite the fact that naloxone treatments blocked *Pb*-induced analgesic-like effect, there was no difference in the survival time relative to the placebo group. Combined, pain processing system contributes little or no input on malaria-related death in mice. Also, evaluation of basic secretory and metabolic activities of the whole brain cells showed that protein level, noradrenaline and activities Na^+^ – K^+^ ATPase and Ca^2+^ – Mg^2+^ ATPase were not significantly different in the *Pb-*infected mice compared to the intact mice. The level of serotonin was, however, increased in the brain of *Pb-*infected mice, although this was not significantly different compared to intact animals but it suggested possible elevation in the biosynthesis of this neurotransmitter. The action of serotonin in the pain modulatory system was claimed to be either attenuation or facilitation of pain signalling (Rahman et al. 2011; Viguier et al. 2013) and either effect can be abolished with serotonin receptor antagonists. Based on the activity of oxidative enzymes evaluated in the plasma, infected animals appeared to have decreased antioxidant enzymes activities compared to the intact mice. Also, total plasma protein and noradrenaline levels were not statistically different in the *Pb-*infected mice relative to intact animals. Again, plasma serotonin was increased in the *Pb-*infected mice compared to intact animals. The increased plasma serotonin might possibly be from lysed platelet cells because estimated platelet cell count was significantly decreased in the *Pb-*infected mice compared to the intact mice.

In conclusion, the present studies attempted to elucidate the influence of host-*Pb* interactions on pain sensitivity and its contribution to malaria-related death. The study showed that the nociceptive processing system of malaria-infected mice has little or no input in malaria-related mortality, and *Pb-*induced malaria elicited analgesic-like effect (decreased pain-related behaviours to noxious stimulus) during critical stage in the host animals. The parasites capacity to interfere with pain sensation appeared to be orchestrated via µ-opioid, α_2_ and 5HT2A receptor as blockage of these receptors elicited increased pain-linked behaviours (at early and/or late phase of formalin test) to noxious stimulus in *Pb-*infected Swiss mice. Owing to all these evidence-based observations, animal model for malaria may not be suitable for investigating malaria-related pain as reported in humans.

## Figures and Tables

**FIGURE 1-A1:**
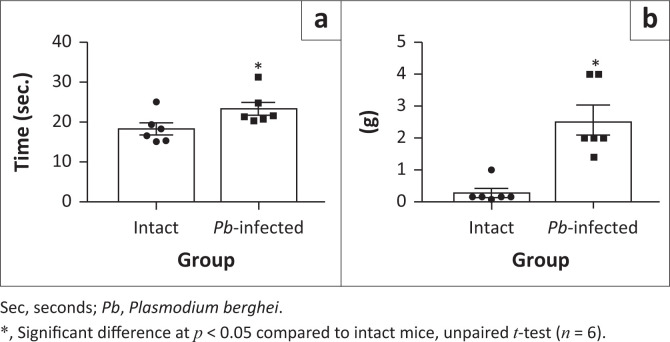
Response thermal and mechanical noxious stimuli. (a) Latency to withdraw paw during cold plate test. (b) Withdrawal threshold in Von Frey test.
